# A Method for the Spatial Interpolation of EEG Signals Based on the Bidirectional Long Short-Term Memory Network

**DOI:** 10.3390/s24165215

**Published:** 2024-08-12

**Authors:** Wenlong Hu, Bowen Ji, Kunpeng Gao

**Affiliations:** 1 The College of Information Science and Technology, Donghua University, Shanghai 200051, China; 2222085@dhu.edu.cn; 2 The Unmanned System Research Institute, Northwestern Polytechnical University, Xi’an 710060, China; bwji@nwpu.edu.cn

**Keywords:** BCI, electroencephalogram, BiLSTM, high-density EEG, motor imagery

## Abstract

The precision of electroencephalograms (EEGs) significantly impacts the performance of brain–computer interfaces (BCI). Currently, the majority of research into BCI technology gives priority to lightweight design and a reduced electrode count to make it more suitable for application in wearable environments. This paper introduces a deep learning-based time series bidirectional (BiLSTM) network that is designed to capture the inherent characteristics of EEG channels obtained from neighboring electrodes. It aims to predict the EEG data time series and facilitate the conversion process from low-density EEG signals to high-density EEG signals. BiLSTM pays more attention to the dependencies in time series data rather than mathematical maps, and the root mean square error can be effectively restricted to below 0.4μV, which is less than half the error in traditional methods. After expanding the BCI Competition III 3a dataset from 18 channels to 60 channels, we conducted classification experiments on four types of motor imagery tasks. Compared to the original low-density EEG signals (18 channels), the classification accuracy was around 82%, an increase of about 20%. When juxtaposed with real high-density signals, the increment in the error rate remained below 5%. The expansion of the EEG channels showed a substantial and notable improvement compared with the original low-density signals.

## 1. Introduction

A brain–computer interface (BCI) establishes a direct communication channel between the human brain and an external device, enabling the control of external devices through neural signals [1,2]. Its basic components include signal acquisition systems, signal processing systems, decoding models, and output devices for the execution of commands. The synergy of these components ensures the conversion of signals from the brain to external devices via the BCI. Currently, there are two main methods of collecting brain signals: invasive and non-invasive methods [3,4].

Invasive methods of signal acquisition require subjects to undergo a craniotomy surgery, during which electrode arrays are implanted into the cerebral cortex to establish direct contact with the neurons, generating electroencephalogram (EEG) signals [1,5]. This method enables the recording of fine neural activity, ensuring a high signal-to-noise ratio and the accuracy of the EEG signals. The collected EEG signals are highly reliable. However, the insertion of external metal electrodes into the brain requires a high level of surgical skill, involves significant surgical risks, and may lead to complications such as infection and bleeding. Due to these limitations, the use of invasive methods is highly restricted. Non-invasive methods, on the other hand, do not require surgical intervention [6]. They directly detect EEG signals on the surface of the scalp using external sensors, thus avoiding surgical risks and complexity. This greatly expands the usability of non-invasive electrodes compared to invasive ones. However, due to interference from the skull and other factors, the quality of the signals collected by non-invasive electrodes is relatively lower and they contain significant noise. To address this issue, various methods are employed in the design of non-invasive electrodes to improve the quality of EEG signals [7,8,9,10,11], such as reducing the impedance and enhancing the signal-to-noise ratio.

It is undeniable that the density of EEG signals is relatively low, despite various improvements in the design of external electrodes. The more signals that are collected, the more information is gathered, leading to higher accuracy in decoding EEG activity [2]. Several studies have shown that as the number of electrodes in EEG devices increases, there are more adjustable parameters, resulting in better control over the quality of the obtained signals [12,13,14]. However, due to the limited surface area of the human cerebral cortex, it is not possible to accommodate an excessive number of electrodes indefinitely [15]. Therefore, the signals collected cannot cover the entire head and are relatively low-quality EEG signals. Moreover, the EEG signals collected from the cerebral cortex are not signals produced by individual neurons [16]. After passing through multiple layers of transmission, the signals are the result of the superposition of multiple neurons. Additionally, although different regions of the brain correspond to different functions, these regions are interconnected, and the brain is not an isolated entity [17].

In scenarios where EEG data are missing from certain electrodes, interpolation is commonly employed to reconstruct the absent electrode signals. The conventional approach to this involves extracting data from neighboring electrodes at the same time instant and utilizing averaging or other mathematical relationships to estimate the missing values. The majority of previous works have adhered to this methodology, with their primary contributions lying in the refinement and iteration of the mathematical functions used [18,19,20]. These studies have sought to improve the interpolation of missing electrode data by employing more sophisticated mapping relationships.

With the advancement of deep learning, its application to EEG has brought new opportunities and challenges to the development of BCI technology [21,22]. Deep learning can uncover relationships and features within data without the need for manual feature extraction algorithms. Additionally, deep learning has shown excellent performance in tasks such as classifying experiments [23,24,25,26], thus aligning well with the relationships among data collected from different electrodes in EEGs. Surprisingly, deep learning is also proficient in handling long-time-series data, which is precisely the nature of EEG signals [27,28,29]. If we consider the signals collected from different electrodes as features or dimensions, this lays the foundation for deep learning to process EEGs effectively [30].

Deep learning provides diverse models for time series analysis. The 1D-CNN captures the local temporal dynamics but falls short in long-range sequence prediction. Deep residual networks, with their residual connections, effectively address the vanishing gradient issue, requiring tuning for optimal performance. LSTM networks are adept at learning long-term dependencies and are sensitive to the hyperparameter settings. GRUs offer a more efficient alternative with fewer parameters, albeit with some loss in capabilities. Following a comprehensive assessment, we selected the BiLSTM model for its bidirectional processing, enhancing the robustness and predictive accuracy in time series analysis.

Motor imagery is an important EEG paradigm aimed at eliciting corresponding motor imagery activity in the brain by providing cues [31,32,33]. Today, motor imagery has expanded beyond a simple experimental task and has been applied in various fields, such as entertainment gaming and health recovery [34,35,36,37]. Motor imagery is a sophisticated type of EEG activity, where individual electrodes record fluctuations in the electrical potentials of the cerebral cortex [38]. For instance, electrodes like C3/C4 are highly sensitive to motor imagery and exhibit corresponding ERD phenomena. However, relying solely on the analysis of individual electrodes has its limitations [39,40]. EEG signals constitute long-time-series data, and, in cases involving multiple electrodes, they contain rich spatial characteristics. Incorporating more information into the data analysis can address the issue of insufficient information in EEG tasks [31,32,33,34,35,36,37,38,39,40,41,42].

Due to various interfering factors, a common issue in most EEG experiments is the inadequacy of the EEG channels. By incorporating more information into the data analysis, it becomes possible to better analyze EEG experiments and explore the human brain [41,42]. To address this issue, we employed deep learning techniques, specifically applying BiLSTM to long-time-series data. We utilized the temporal and spatial features present in EEG data, reconstructing the data for motor imagery classification tasks. This approach allows us to transform a low-density EEG signal into a high-density one, thereby enhancing the analysis of EEG experiments.

## 2. Materials and Methods

### 2.1. Interpolation Principle

EEG signals, as time series data, have multiple characteristics: they reflect dynamic changes in brain activity, including spontaneous EEGs of different frequencies and EEG responses related to specific events. These signals have rich temporal characteristics, involving changes in multiple time scales, and may contain dynamic patterns related to cognitive tasks, emotional states, or neurological disorders. The LSTM network can effectively process EEG data. Firstly, the LSTM network can capture long-term dependencies in time series data through its internal gating mechanism, thereby better understanding dynamic changes in brain activity. Secondly, LSTM networks can automatically learn temporal feature representations from EEG data, thereby more effectively extracting relevant information. In addition, LSTM networks identify and predict dynamic pattern changes in EEG data through historical information, thus having great potential for diagnostic, classification, or prediction tasks. Finally, the LSTM network is capable of processing EEG data with different time scales, thereby more accurately understanding complex, dynamic changes in brain activity.

**Figure 1:**
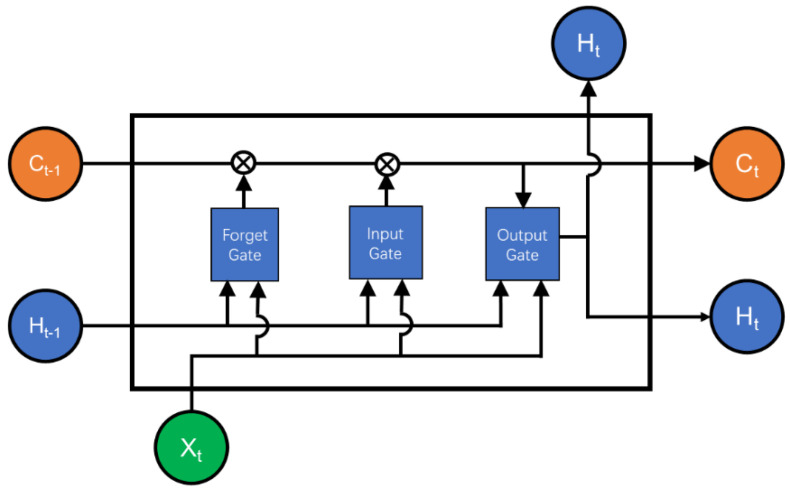
The structure of the LSTM unit.

The unit structure of LSTM is shown in Figure 1, and the core structure in LSTM, denoted as $C_{t}$, plays a pivotal role in transmitting and preserving long-term dependencies across the network. Throughout the propagation process, $C_{t}$ undergoes continuous modifications facilitated by gating units. On the other hand, $h_{t}$ serves dual purposes: it contributes to both the loss calculation of the current model and the gradient calculation for subsequent layers.

(1) $$\begin{array}{c} f_{t}=\sigma \left(W_{f}\cdot \left[h_{t-1},x_{t}\right]+b_{f}\right) \end{array}$$

(2) $$\begin{array}{c} i_{t}=\sigma \left(W_{i}\cdot \left[h_{t-1},x_{t}\right]+b_{i}\right) \end{array}$$

(3) $$\begin{array}{c} o_{t}=\sigma \left(W_{o}\cdot \left[h_{t-1},x_{t}\right]+b_{o}\right) \end{array}$$

Formulas (1)–(3) delineate the compositions of three gate units, each sharing a similar structure. Each gate incorporates the previous state *h_t_*_−1_ and couples it with the current data information *x_t_*. The amalgamation of these two inputs undergoes processing by the weight matrix *W* and bias *b*, followed by the application of the sigmoid function to yield a value ranging from 0 to 1. When the gate control switch is set to 0, information transmission is inhibited; conversely, when set to 1, all information is permitted to pass through. Consequently, the forget gate regulates the degree of old information to be discarded, the input gate governs the influx of new information, and the output gate determines the amalgamated output of both new and old information. This mechanism facilitates the flow of data through the network in a manner akin to memory.

(4) $$\begin{array}{c} {\tilde{C}}_{t}=tanh\left(W_{c}\cdot \left[h_{t-1},x_{t}\right]+b_{c}\right) \end{array}$$

(5) $$\begin{array}{c} C_{t}=f_{t}\cdot C_{t-1}+i_{t}\cdot {\tilde{C}}_{t} \end{array}$$

(6) $$\begin{array}{c} h_{t}=o_{t}\cdot tanh\left(C_{t}\right) \end{array}$$

Formulas (4)–(6) represent the information update process within the LSTM network. As depicted in Equation (4), despite its structural resemblance to the gate unit, the activation function, denoted as *tanh*, is employed to define ${\tilde{C}}_{t}$ for distinction purposes. Here, ${\tilde{C}}_{t}$ denotes candidate cells, signifying the memory of cell initialization. Following the establishment of the initial memory, subsequent memory cells $C_{t}$ are combined with it to integrate new memories. This process necessitates the utilization of forget gates and input gates to regulate information screening, as outlined in Formula (5). Finally, during the output stage, the cell state undergoes filtration via the output gate to determine which information will be propagated to the hidden state $h_{t}$ in the subsequent time step.

### 2.2. BiLSTM

**Figure 2:**
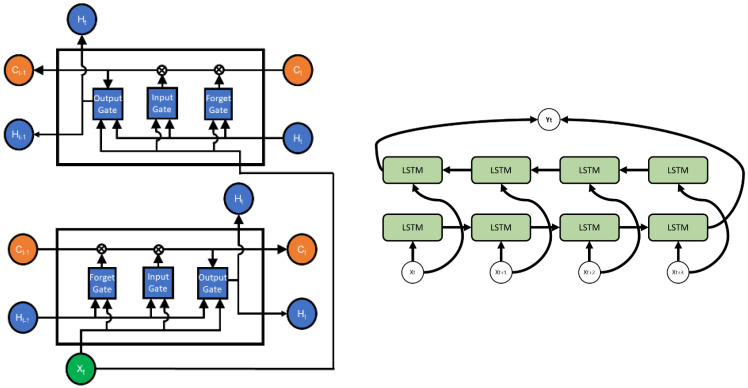
The structure of the LSTM unit and a simple BiLSTM model.

**Figure 3:**
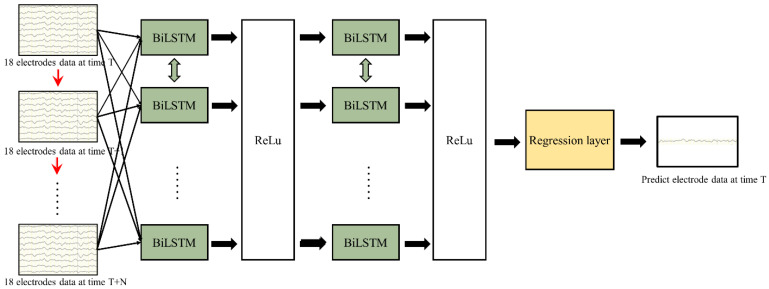
The BiLSTM network structure diagram.

The preceding section has delineated the pertinent principles underlying LSTM, and further discussion of these principles will be omitted herein. The network architecture adopts BiLSTM, as shown in Figure 2, which includes a double-layer LSTM stacked in both the forward and reverse directions This stacking configuration mitigates BiLSTM’s reliance on immediate data points and retrieves the connections between the data from the bidirectional propagation of time series, enhancing the predictive abilities; this is especially beneficial in processing input data, such as EEG signals characterized by long durations and signal complexity The schematic representation of the network structure is depicted in Figure 3.

Figure 3 illustrates the architecture of the BiLSTM network utilized in this study. We employed two stacked BiLSTM layers to process both historical and future time series data, ensuring the robust capture of the data connections within EEG signals. To ensure the correct functioning of the network model with EEG data as input signals, we undertook the following preprocessing steps.

(1) Initially, we partitioned the EEG data into two feature dimensions: the temporal and spatial dimensions. The spatial dimension was segmented into distinct electrode data, while the temporal dimension was organized into different time points.
(2) Spatial information was aggregated into comprehensive features, denoted as *x_t_*, enabling the processing of multiple electrode data with a single-time input.
(3) The input data were sequenced chronologically along the time dimension (*t*/*t* + 1/*t* + 2), with each moment’s data features being multidimensional and inclusive of multiple electrode data.

Considering the vast spatiotemporal features inherent in EEG data, we opted to segment the data to alleviate the processing burden on the computers. Each experiment comprised ten complete rounds of motor imagery experiments, with eight rounds allocated for training and two rounds reserved for testing to validate the model’s performance.

### 2.3. Tools and Parameters

As our platform for data processing, MATLAB2020a, a software program renowned for its high-performance numerical computation capabilities, is particularly well suited to algorithm development. The Bilateral Long Short-Term Memory (BiLSTM) algorithm, central to our experiment, leverages MATLAB’s integrated Neural Network Toolbox. This toolbox facilitates user interaction and ensures computational efficiency, yielding results of exceptional quality. Furthermore, the EEGLAB toolbox significantly augments MATLAB’s capabilities in the domain of EEG signal processing. It offers a streamlined process for EEG data manipulation, requiring only the importation of EEG data into EEGLAB for comprehensive data processing. This unique advantage positions MATLAB as an indispensable tool in our research, streamlining the workflow and enhancing the accuracy of our EEG signal analysis.

(1) Batch Size: The batch size is a hyperparameter in deep learning that represents the number of samples processed in forward and backward propagation in the BiLSTM algorithm. Its size determines the generalization ability and training stability of the model, as well as the memory consumption requirement. Considering the sensitivity of EEG data processing and the computational efficiency of the hardware, we chose the batch size to be within the range of 80–120 in the dataset with a frequency of 250 Hz.
(2) Hidden Units: Hidden units are another fundamental hyperparameter in neural networks, and their quantity also determines the model’s performance. Due to the unique gate mechanism of hidden units in BiLSTM, the more models there are, the better the fit to the data. However, there is also a risk of overfitting. Due to the bidirectional transmission effect, the number of selected hidden units is 120 and 128, respectively, and a double-layer BiLSTM is used for stacking.
(3) Sequence Length: The sequence length determines the number of time steps processed by each forward and backward propagation. If this value is set too high, although it can capture long-range memory relationships, it increases the computational requirements and the risk of gradient vanishing. On the contrary, the computational requirements are small, but the utilization rate of time memory before and after is also meager. This article sets the sequence length to 120–180, which is about 0.5 s of time memory in the 250 Hz dataset.

### 2.4. Data Preprocessing

(1) Step 1—Identification of artifacts: Manually inspect the data and label the identified artifacts, or set a threshold through EEGLAB (v2023.0), so that signals outside the threshold are marked.
(2) Step 2—Artifact removal: By checking the frequency components through the marked artifact parts and using a filter to filter out signals in specific frequency bands, the pure signal after removing the artifacts can be obtained. Alternatively, independent component analysis (ICA) can be used to decompose the EEG signals into independent components and identify and remove components related to artifacts.
(3) Step 3—Choose baseline window: Usually, a time window before stimulation is chosen as the baseline period. For example, if the stimulus appears at 0 ms, a time period of [−200 ms, 0 ms] can be selected as the baseline window.
(4) Step 4—Baseline correction: Calculate the average value of each signal within the baseline window and subtract the baseline mean of the corresponding channel from the recorded EEG signal.
(5) Step 5—Dataset splitting: To ensure temporal data continuity, we performed 5-fold cross-validation on our dataset, using 8 out of 10 experimental rounds for training and the remaining 2 for testing.

### 2.5. Dataset

The dataset utilized in this study is sourced from dataset 3a of the BCI Competition III (https://www.bbci.de/competition/iii/desc_IIIa.pdf, accessed on 1 October 2022). It comprises EEG data from a four-class motor imagery experiment involving three subjects. The EEG data were recorded at a sampling frequency of 250 Hz and encompassed a total of 60 electrodes. For visual artifact processing, source derivatives based on the center and four nearest-neighbor electrodes were calculated, without considering artifacts in the boundary electrodes.

**Figure 4:**
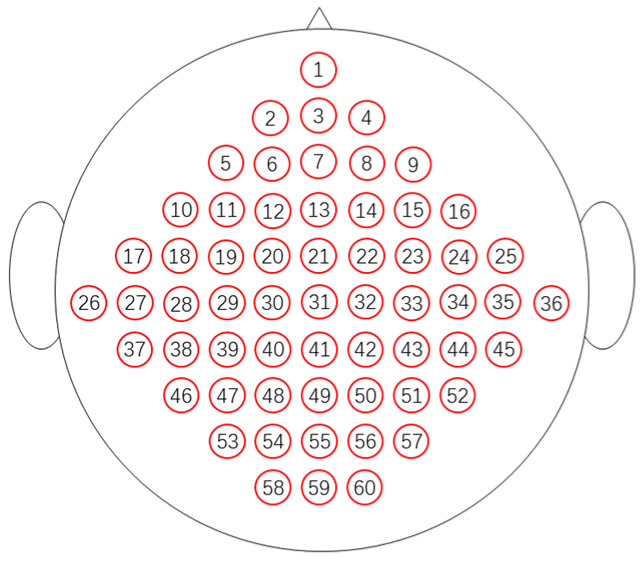
Distribution map of electrode positions.

Figure 4 provides a rough schematic of the electrode positions used. According to the rules of the international 10–20 system clues, this article uses tables (see Table 1) to illustrate the channels used in the dataset.

In the initial 2 s interval of each trial, no action was required from the subjects. Subsequently, at the 2 s juncture, a prompt manifested in the form of an auditory cue, concomitant with the appearance of a stationary crosshair on the screen, served as an initiation signal for the subjects to commence the experiment. Following this cue, the computer interface presented a set of four directional arrows, each indicative of motor tasks associated with left-hand, right-hand, foot, and tongue movements, respectively. Subjects were instructed to engage in mental rehearsal corresponding to the indicated motor actions delineated on the display. Each trial spanned a duration of 4 s, culminating in a brief interlude allocated for recuperative rest before transitioning to the subsequent trial iteration.

### 2.6. Locations of Electrodes

**Figure 5:**
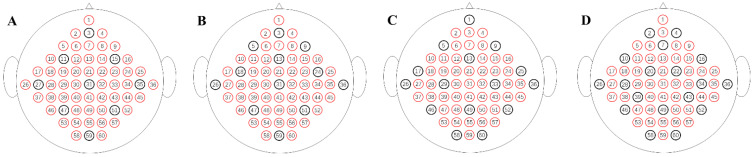
Four initial electrode combination methods (**A**–**D**).

**Figure 6:**
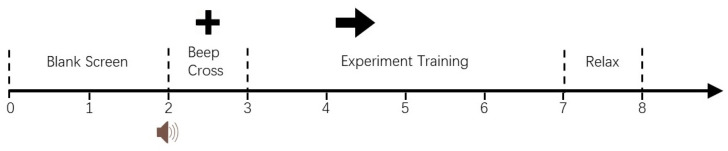
Timing of the imaginary movement paradigm.

As mentioned in the previous sections, the locations of the electrodes are a crucial aspect, as the foundation of the upcoming experiment. For this purpose, meticulous electrode selection within the training dataset was of paramount significance in this experimental setup. Despite the pivotal role of the region of interest (ROI) in motor imagery experiments, to ensure the generalizability of the reconstructed data, the electrode sampling in this study ensured comprehensive coverage across the entire brain. Employing a left–right symmetrical selection approach, four distinct methods were employed, encompassing electrode configurations of 9, 12, 15, and 18 (see Figure 5). The outcomes of these selections are delineated in Figure 6.

## 3. Results

### 3.1. Predicted Data

Four electrode selection methods (utilizing 9, 12, 15, and 18 electrodes) were implemented to enhance the low-density signals. Subsequently, the BiLSTM network was employed to conduct signal super-density processing on various combinations, with the resultant outcomes depicted in the following figure. Additionally, this study delved into alternative approaches for the generation of “pseudo-super-density” signals, including the mean method and cubic spline interpolation, which were utilized for comparative analysis with the proposed methods, as described in the next section.

Figure 5 displays the data results for a single electrode obtained using the BiLSTM model for four-electrode combinations. To facilitate comparison, we have distinguished the estimated values from the true values using blue and red, respectively. It is evident that the root mean square error of the results predicted using BiLSTM on the training set remains within 0.6, considering the data’s floating range of −40 to 30. Furthermore, as the initial number of electrodes increases, the error on the training set continuously decreases, reaching a minimum of 0.2087.

Similarly, based on the errors observed on the test set, it is apparent that the predicted data and the true values maintain a high degree of consistency, with noticeable anomalies only observed in some specific areas (sudden spikes in the data). Analogous to the training set, as the initial number of electrodes increases, the error of these outliers also diminishes. This phenomenon can be attributed to the increased number of sample electrodes, which enables the acquisition of more EEG information and enhances the connectivity between the electrodes.

**Figure 7:**
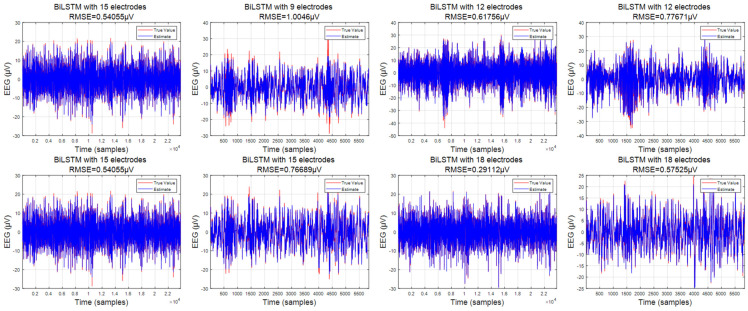
The error of the proposed BiLSTM; the left column is the training set, and the right column is the test set.

Additionally, we conducted a detailed comparison of the error magnitude in predicting the same electrode (electrode 21) between 9 electrode combinations and 18 electrode combinations. In Figure 7, we present the results for both the training and testing sets, which distinguish between the data volumes in each set. It is evident that, on the training set, both combinations yield results that are highly consistent with the original real EEG data, achieving root mean square errors of around 0.5 and 0.2, respectively.

**Figure 8:**
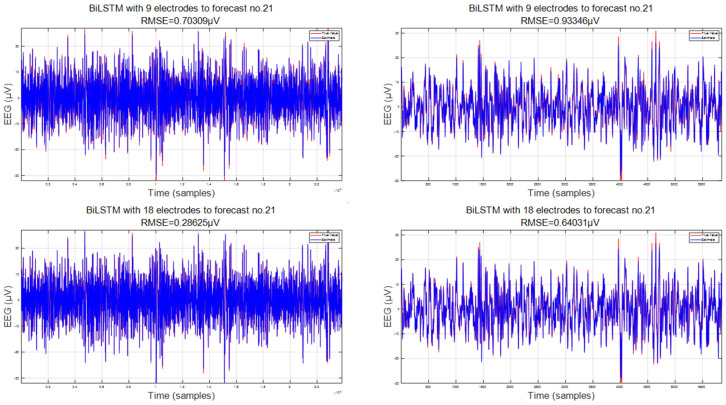
Comparison of two electrode combinations: 9- and 18-electrode combinations.

Furthermore, upon comparing the test sets of the two combinations in Figure 8, it becomes evident that the effect of the 18-electrode combination is highly significant on the test set. This is attributed to the fact that EEG data represent a time series containing a substantial amount of spatial information. The spatial information encapsulated within the 18-electrode combination effectively bridges the connections between different electrodes, thereby leading to improved predictions. The root mean square error has also decreased from 1.0 to 0.6. At this level, the error effect on the test set with 18-electrode combinations can be compared to the error on the training set with nine-electrode combinations. Moreover, the average width of the confidence interval for the combination of nine electrodes is 1.0511 μV, with a coverage rate of 94.4981%. However, the average width of the confidence interval for the combination of 18 electrodes is 0.2076 μV, with a probability of 95.3614%.

**Figure 9:**
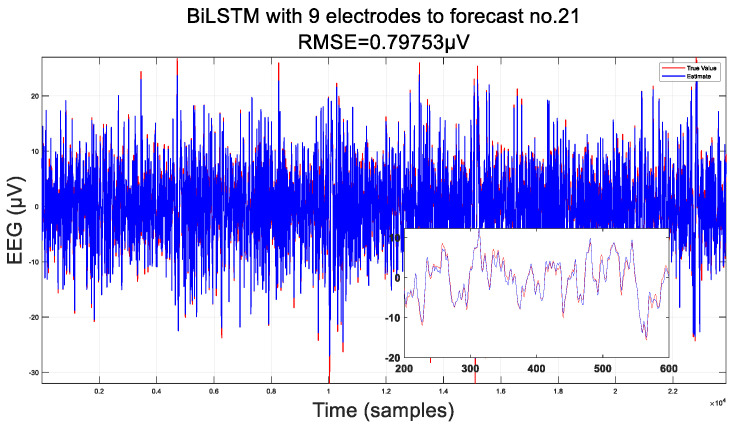

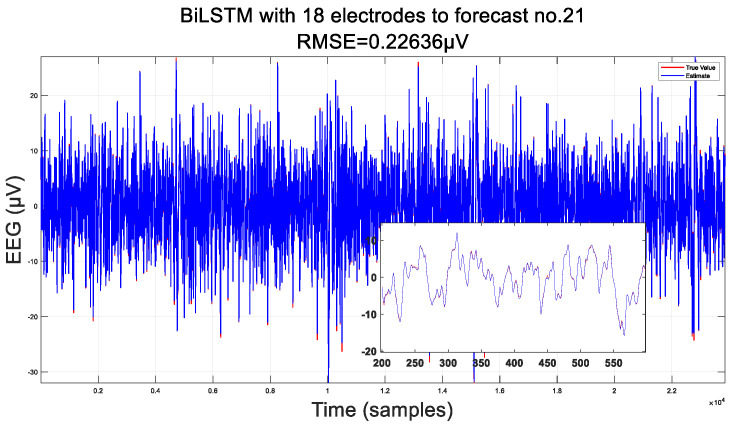
Comparison of detail magnification for two electrode combinations: 9- and 18-electrode combinations.

Upon the careful examination of the enlarged comparison presented in Figure 9, it becomes evident that the interpolation data derived from utilizing only 18 electrodes closely approximate the true values. This observation challenges the assumption that fewer electrodes are inherently disadvantageous. Instead, it underscores the significance of achieving the uniform distribution of the electrodes across the brain, which is crucial in obtaining accurate and reliable EEG data. The quality of EEG data interpolation is not solely determined by the number of electrodes but is also significantly influenced by their spatial arrangement. A well-distributed array of electrodes, even with a smaller count, can yield interpolation results that are more representative of the actual neural activity.

### 3.2. Comparison of Interpolation Methods

**Figure 10:**
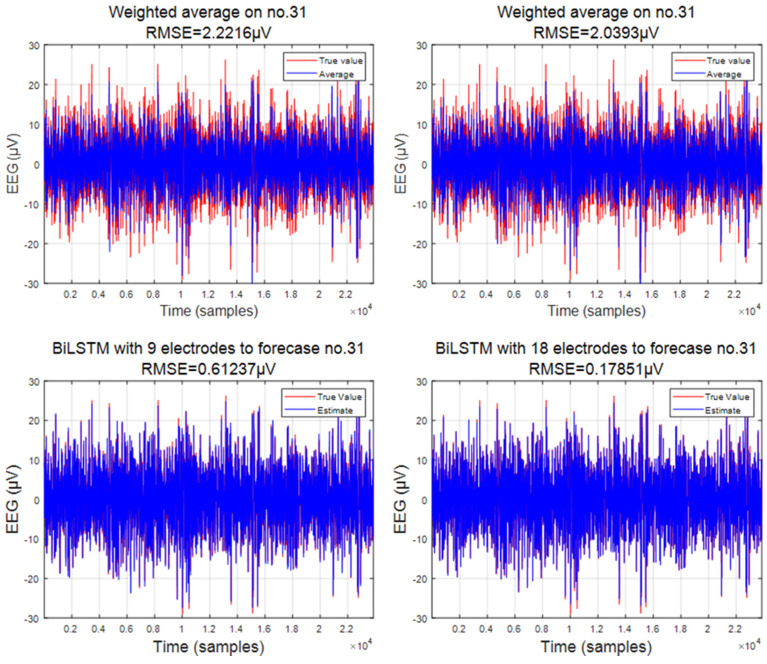
Comparison of weighted average and BiLSTM.

**Figure 11:**
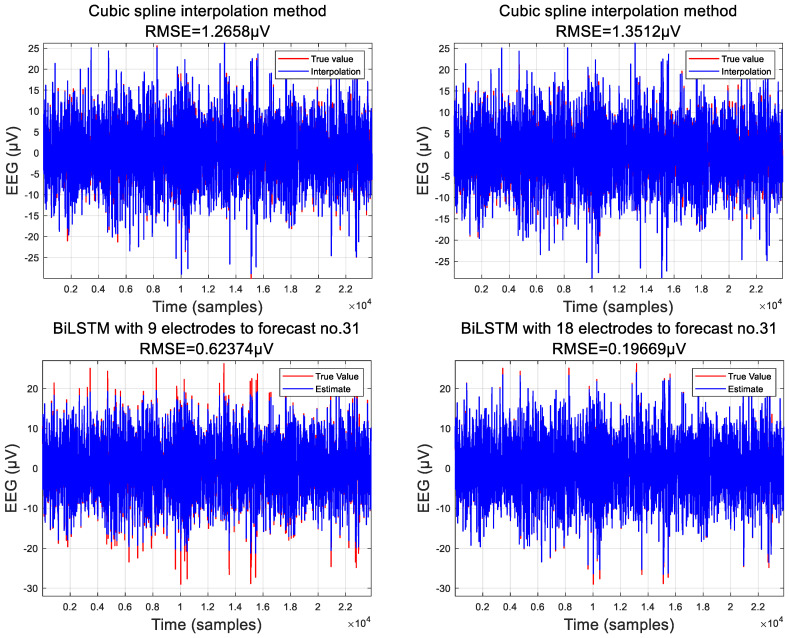
Comparison of cubic spline and BiLSTM.

Traditional interpolation methods often lack consideration of the underlying connections within the data, resulting in abrupt transitions. These methods typically employ simplistic functions or stacking techniques to fill in missing data points. While they may yield satisfactory results in certain cases, their efficacy is often questionable. In this context, we present two interpolation methods for comparison: weighted average and cubic spline interpolation. See Figure 10 and Figure 11.

The weighted average method computes a numerical average based on the electrode combinations available. Here, the weights of different electrodes are determined by their distances from the predicted electrode. Closer electrodes carry higher weights, while distant ones have lower weights. Specifically, we project the existing electrodes onto a plane and derive the weights by calculating the distances between them on a two-dimensional plane. The weights are assigned as the reciprocal of the distance or the reciprocal of the square of the distance. The performance of the weighted average method in predicting the data for electrode 31, utilizing 18 electrodes as the sample average, is presented herein. It is evident that, whether employing the reciprocal distance or the reciprocal square of the distance, significant errors are observed, with root mean square errors of 2.9947 and 2.7752, respectively. Conversely, leveraging BiLSTM for the prediction of electrode number 31 yields notably superior results on both the training and testing sets, with a root mean square error consistently maintained within 0.41. It is apparent that the performance achieved through the weighted average method is neither satisfactory nor plausible. 

The weighted average interpolation method has confidence intervals of 8.0799 μV, and 7.9946 μV, for the two interpolation methods, respectively. With such a large confidence interval, their coverage rate also reaches around 93%. Similarly, we used the BiLSTM method to obtain confidence intervals of 0.2475 μV, and 0.864 μV, on electrode 31, with confidence interval coverage rates of 93.8756% and 94.8991%, respectively.

Based on the fundamental principle of cubic spline interpolation, our choice of electrode coordinates can only vary in one direction. Therefore, our electrode selection cannot be arbitrary. Here, we have modified the selection of the sample electrodes. To predict electrode number 31, we selected sample electrodes along the horizontal or vertical line where electrode number 31 is located. We used the data from these sample electrodes as known points and the distances between the electrodes as the basis for interpolation. This allowed us to obtain a cubic spline function and consequently the data for electrode number 31. Although cubic spline functions can simulate the trend of data variation on the same line, the errors are still significant, with a root mean square error (RMSE) exceeding 1.8. This is because the interpolation point selection of cubic splines results in the loss of many spatial features of the sample electrodes, merely fitting the data based on the trend of data variation. There is still a considerable gap compared to the results of BiLSTM.

Cubic spline interpolation, distinguished by its specific computational approach, was compared within our study. It demonstrated confidence intervals of 1.3512 μV and 1.2658 μV, with coverage rates of 95.12% and 94.8002%, respectively. While these outcomes may seem promising, the relatively wide confidence intervals suggest a broader range of potential error. For instance, on electrode 31, cubic spline interpolation performed adequately. However, its efficacy in fitting other electrodes could vary significantly, particularly due to the limited electrode count, with the errors potentially peaking at approximately 5.7 μV.

While traditional interpolation methods are prevalent, they often yield higher errors compared to the BiLSTM approach. The transmission of EEG information is not merely a product of processing by individual neurons or a cluster of nearby neurons. The BiLSTM algorithm adeptly captures this complexity, enabling the establishment of connections between even distant electrodes through its sophisticated algorithmic framework. This capability contributes to the superior performance of BiLSTM, particularly in scenarios where the amplitudes of the surrounding electrodes exhibit similarity.

In the context of experimental procedures, the selection of an inappropriate interpolation method can lead to the generation of erroneous data. Such data, when utilized in subsequent analyses and processing, can render the results meaningless. For instance, in the BCI Competition 3a dataset, the utilization of inaccurately interpolated data for motor imagery classification can result in ambiguous outcomes, thereby deviating from the intended purpose of interpolation, which is to enhance the clarity and accuracy of EEG signal analysis.

The BiLSTM method ensures comprehensive data coverage while keeping the errors to a minimum. Utilizing a modest number of electrodes that are uniformly distributed across the scalp, our approach effectively extrapolates to a full 60-electrode array. This is a significant advancement over traditional methods, which are restricted to enhancing a single electrode within a localized region. The mapping relationships in traditional methods are often limited and require iterative adjustments to the initial sample electrodes to achieve any form of meaningful extension.

At its core, traditional interpolation is inherently incapable of scaling from a sparse electrode setup to a denser configuration. In contrast, the BiLSTM method, with its advanced learning algorithms, overcomes this limitation, offering a robust solution for EEG data analysis even when working with a reduced number of electrodes.

### 3.3. Analysis of EEG Signals

The preceding discussion has delineated the specific errors between the actual and predicted values on individual electrodes. However, the evaluation of EEG data extends beyond isolated electrode errors. To assess the cumulative impact of errors, EEG topographic maps are essential in gauging the overall consistency of the data. EEG topographic maps provide a means to visualize brain activity through the spatial aspect of EEG signals. They depict the distribution of the signals on the scalp’s surface, offering intuitive spatial information about the brain’s activity. The activity levels of EEG signals are depicted by the color scheme of the EEG topographic map, with warm or red tones indicating a higher intensity of EEG activity and cool or blue tones indicating a lower intensity.

Following the international standard 10–20 electrode placement system, EEG topographic maps convert electrode data into different color-coded blocks spanning the entire brain region. This transformation process renders initially ambiguous data into a comprehensible EEG topographic map, facilitating improved visualization and interpretation.

**Figure 12:**
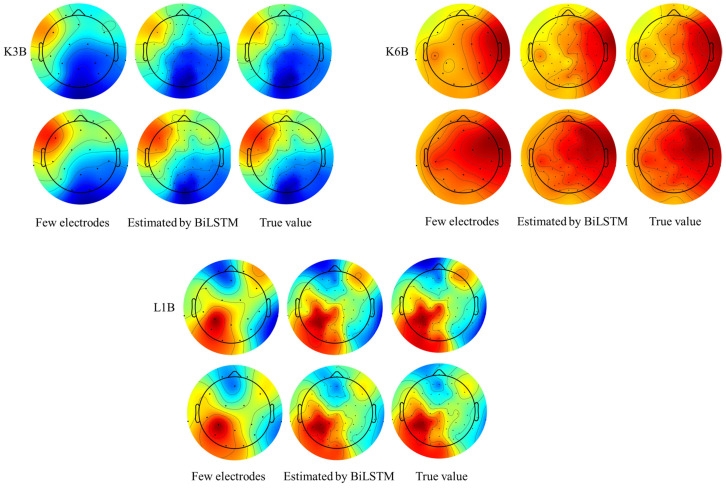
EEG plot; the **left** column shows the data of a few electrodes, the **middle** column shows the estimated data from BiLSTM, and the **right** column shows the true values.

Figure 12 illustrates the EEG topographic map, wherein we horizontally compare the results obtained from 18 electrodes, 60 electrodes generated through BiLSTM, and the original 60 electrodes. Continuous EEG topographic maps of three subjects at different periods are provided, with temporal continuity depicted through vertical EEG topographic maps. It is evident that the colors of the EEG topographic maps in the three figures are largely consistent, and the uniformity of the color blocks underscores the rationality of the data. In other words, the data constructed through BiLSTM are generally coherent in terms of the overall electrode configuration and adequately cover all areas of the cerebral cortex.

To further discern the distinctions among the three scenarios, we include contour lines in the EEG topographic map, which effectively delineate the trends in the cortical EEG data changes. It is evident that with an increase in the number of electrodes, the contour lines in the EEG topographic map also multiply, indicative of the richness of the EEG data. Only when the data volume is sufficient can the evolving features become apparent.

Moreover, upon scrutinizing the EEG topographic map of the 18-electrode combinations, it is notable that the position of the contour lines primarily resides within the boundaries of different color blocks, playing a minimal role in the overall analysis. In contrast, the EEG topographic maps generated using BiLSTM can reveal various changes within the same color block, as observed in the EEG topographic maps of the three subjects. This observation is consistent with real-world scenarios. While inconsistencies may arise at specific points along the contour line or due to fluctuations in data from a single electrode, such occurrences are deemed normal due to the exceedingly complex nature of brain signals. Deep learning algorithms may not predict mutations in specific areas at precise moments.

Nevertheless, when compared to a few combinations of 18 electrodes, BiLSTM exhibits a high degree of consistency with the real data, delivering commendable performance across consecutive moments.

### 3.4. MI Task Evaluation

**Figure 13:**
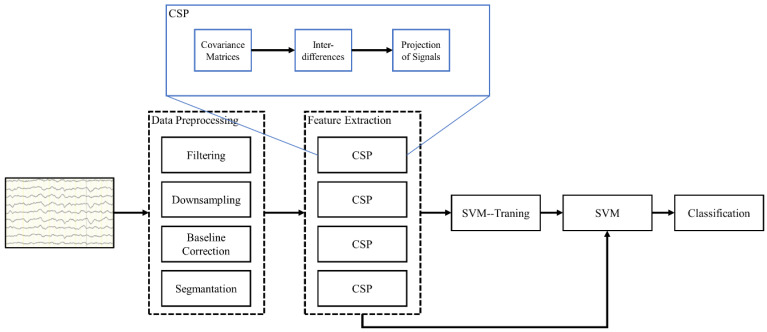
Classification flow chart of MI task.

Motor imagery is a classic experimental paradigm in EEG experiments, where participants need to simulate corresponding motor responses in the brain based on specific prompts. The classification task is based on the excitement levels of different brain regions, which are distinguished by the activity levels of the signals. Therefore, it requires a high density of EEG signals. When the density of the EEG signals is relatively high, the classification effect obtained will be significantly better. The EEG dataset used in this experiment is from dataset 3a of BCI Competition III. As the data processing has already been completed in the previous stage, the classification process requires the use of a Common Spatial Pattern (CSP) for feature extraction in advance, followed by One-Versus-Rest (OVR) to traverse all features for classification determination. To further verify the credibility of the data, we not only compare the predicted 60 electrodes with the original 60 electrodes but also use a few electrodes, namely 18, as a control group for reference. See Figure 13.

Table 2, Table 3 and Table 4 present the outcomes for the four categories of motor imagery across a subset of 18 electrodes, 60 electrodes obtained using BiLSTM, and the original 60 electrodes. The results indicate that expanding from 18 electrodes to 60 electrodes leads to improvements in both the individual and overall classification accuracy.

Specifically, the total classification accuracy for the subset of 18 electrodes is 63% ± 3%. After expansion, the accuracy increases to approximately 83%, marking a 20% improvement over the same period. Moreover, the classification accuracy for individual categories exhibits significant enhancements. For instance, the accuracy for the K3B individuals’ single category increases from 60 ± 3% to 85 ± 3%, representing a substantial improvement. Even the classification accuracy for foot movements elevates from 42.22% to 73.33%. These improvements are consistent across subjects K6B and L1B as well.

**Figure 14:**
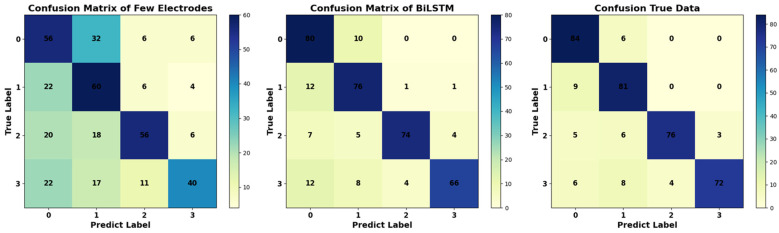
Confusion matrix of three types of data.

Comparing the classification results of the expanded 60 electrodes with the actual 60 electrodes reveals minimal differences in individual categories. For example, the classification results for the two left-handed categories for K3B are 93.33% and 88.89%, while the results for the two tongue categories for L1B are 86.67% and 90%. Even in the challenging-to-distinguish foot categories, the classification results for the two categories for K3B are 76.67% and 77.78%. Figure 14 presents the confusion matrix for the classification results of the three types of data, where the main diagonal represents the number of correctly classified instances for each task class, and darker-colored blocks are employed for differentiation. Observing the matrix, it is evident that significant errors persist in the classification tasks conducted with a limited number of electrodes. Meanwhile, the classification results obtained through BiLSTM closely align with the true values along the main diagonal, indicating the minimal errors between them. This improvement in accuracy can be attributed to the model’s capability to learn the relationships between the electrodes and enhance the spatial density of the EEG data, thereby significantly enhancing the classification performance.

## 4. Discussion

The BiLSTM network model proposed in this article offers a significant advancement over traditional interpolation methods in the realm of EEG data processing. Theoretically, EEG data are derived from diverse sources and exhibit temporal dependencies that are not captured by simple peripheral electrode interpolation [17]. Despite the superficial similarities in the results, the traditional approach overlooks the fundamental temporal dynamics inherent in EEG signals. EEG data are inherently a time series, characterized by dependencies between successive data points. This temporal nature is not adequately addressed by traditional methods, which treat data points as independent entities [40,41,42]. In contrast, BiLSTM is specifically designed to handle such temporal sequences, making it a more suitable tool for EEG data interpolation [27]. This aligns with the emerging trend towards few-electrode interpolation, where the need for extensive electrode arrays is reduced.

When employing ICA for EEG data processing, one frequently encounters the issue of rank dimensionality. This occurs because the use of an average reference can diminish the rank of the EEG dataset, consequently impacting the performance of the ICA. It is typically recommended to apply the average reference only after the ICA has been conducted. However, traditional interpolation techniques, due to their straightforward mathematical mapping of electrode relationships, result in newly interpolated electrode data that remain a linear combination of the original data, thereby reintroducing the rank deficiency issue. Nonetheless, the BiLSTM method effectively circumvents this challenge.

## 5. Conclusions

This paper proposes a dual-layer BiLSTM structured network aimed at expanding a low-density channel EEG dataset into a high-density channel one. Specifically, the RMSE is reduced from 0.9 to 0.3, indicating a substantial improvement in data quality from BiLSTM over traditional interpolation methods. This enhanced accuracy is not only reflected in the interpolated data themselves but also in the four-class imaginary movement classification task. Our experiments show that the accuracy of the four-class motor imagery task has been notably enhanced by 25%. While most studies have focused on various methods of processing data to achieve better results, this paper argues that the fundamental issue in research related to brain activity lies in the limited scalp coverage and scarcity of EEG data. By leveraging the interrelation of information in relevant brain regions using deep learning, we obtain more data, specifically reflecting increased scalp coverage, making EEG research more effective.

However, we also recognize the computational challenges associated with algorithmic interpolation. This process demands substantial computational resources, which limited the volume of data utilized in this study. Additionally, the variability in EEG data across different tasks hinders the model’s generalizability. These factors underscore the need for further research and development.

Looking ahead, as the computational capabilities continue to advance, we anticipate that algorithmic interpolation will be able to process data from a minimal number of electrodes directly through the BiLSTM model. This will enable the achievement of high-quality data performance, marking a significant step forward in the field of EEG data analysis.

## Figures and Tables

**Table 1:** Index of the electrodes.

1:Nz	2:Fp1	3:Fpz	4:Fp2	5:AF7	6:AF3	7:AFz	8:AF4	9:AF8	10:F7
11:F5	12:F3	13:Fz	14:F4	15:F6	16:F8	17:FT7	18:FC5	19:FC3	20:FC1
21:FCz	22:FC2	23:FC4	24:FC6	25:FT8	26:T9	27: T7	28:C5	29:C3	30:C1
31:Cz	32:C2	33:C4	34:C6	35:T8	36:T10	37:TP7	38:CP5	39:CP3	40:CP1
41:CPz	42:CP2	43:CP4	44CP6:	45:TP8	46:P7	47:P5	48:P3	49:Pz	50:P2
51:P4	52:P6	53:PO7	54:PO3	55:POz	56:PO4	57:PO8	58:O1	59:Oz	60:O2

**Table 2:** Four classification experiments on imaginary task with 18 electrodes.

	Left Hand	Right Hand	Tongue	Foot	Total
K3B	57.78%	64.44%	64.44%	42.22%	61.75%
K6B	86.67%	60%	57.33%	43.33%	67.48%
L1B	60%	76.67%	66.67%	46.19%	62.53%

**Table 3:** Four classification experiments on imaginary task with 60 electrodes by BiLSTM.

	Left Hand	Right Hand	Tongue	Foot	Total
K3B	88.89%	84.44%	82.22%	73.33%	82.22%
K6B	84.44%	84.44%	86.66%	77.78%	83.33%
L1B	86.67%	80%	90%	73.3%	82.5%

**Table 4:** Four classification experiments on imaginary task with 60 true electrodes.

	Left Hand	Right Hand	Tongue	Foot	Total
K3B	93.33%	88.89%	82.22%	80%	86.85%
K6B	93.67%	93.33%	83.33%	76.67%	87.44%
L1B	92.67%	90%	86.67%	83.3%	89.16%

## Data Availability

The original contributions presented in the study are included in the article, further inquiries can be directed to the corresponding author.
